# The effect of prior cancer on non‐small cell lung cancer trial eligibility

**DOI:** 10.1002/cam4.4049

**Published:** 2021-06-18

**Authors:** Michael Herman, Zhihui Liu, Frances A. Shepherd, Natasha Leighl, Geoffrey Liu, Penelope A. Bradbury

**Affiliations:** ^1^ Princess Margaret Cancer Centre University of Toronto Toronto ON USA; ^2^ Dalla Lana School of Public Health University of Toronto Toronto ON USA

**Keywords:** clinical trials, eligibility, NSCLC, prior cancer

## Abstract

**Objectives:**

Approximately 20% of patients diagnosed with non‐small cell lung cancer (NSCLC) have a history of prior (non‐lung) cancer. Patients with prior cancer are frequently excluded from clinical trials. We aimed to assess the potential impact of prior cancer on commonly used clinical trial endpoints.

**Materials and Methods:**

Clinical trials of systemic therapy for incurable NSCLC from clinicaltrials.gov were reviewed to determine the frequency of exclusion on the basis of prior cancer. A cohort of patients with incurable NSCLC and prior cancer, treated with first‐line systemic treatment at our institution were reviewed as a surrogate clinical trial population. A list of priori events was developed to capture the potential for prior cancer to negatively affect clinical trial conduct or endpoints. The proportions of patients that developed an outcome were assessed.

**Results:**

Among trials registered on clinicaltrials.gov, 66% listed prior cancer in the eligibility criteria, and of these 35% excluded patients with prior cancer in the last 5 years. Of NSCLC patients treated with systemic therapy at Princess Margaret Cancer Center, 20% had prior cancer, of these, breast (20%) and prostate (19%) were the most common malignancies. Median time between prior cancer and NSCLC was 82 months. Median survival was 20 months. For patients without evidence of active prior cancer at baseline, and not on active therapy for prior cancer, no patients had evidence of a recurrence of prior cancer during the treatment and follow‐up for the NSCLC, nor died from prior cancer. However, two patients developed new primaries.

**Conclusions:**

A history of prior cancer has a low likelihood of impacting clinical trial endpoints in patients with incurable NSCLC, if not active or requiring treatment. These findings should be validated in larger data sets.

## Graphical abstract

Clinical trials frequently exclude patients with prior malignancy from participation. ASCO guidelines recommend increased inclusion of patients with prior malignancies in trials. We have evaluated the frequency that patients with prior malignancies are excluded from clinical trials and evaluated the potential impact of prior malignancy in a cohort of patients with advanced non‐small‐cell lung cancer treated with systemic therapy

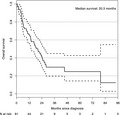

## 1 INTRODUCTION

Only 3% of patients with a diagnosis of cancer are enrolled on clinical trials in the United States.^1^ This is despite estimates that up to 70% of patients are very willing to participate in trials,^2^ suggesting that multiple barriers exist to enrollment. Prohibitive clinical trial eligibility criteria have been long cited as one of these barriers,^3^, ^4^, ^5^ and have led to initiatives to make clinical trial eligibility criteria more inclusive.^6^, ^7^

Lung cancer is the leading cause of cancer‐related death.^8^ While there have been substantial improvements in the survival of patients diagnosed with lung cancer, only a minority of patients are cured, highlighting the pressing need for ongoing research and the evaluation of new treatments to reduce the public health burden from this disease.

In 2017, the American Society of Clinical Oncology and the Friends of Cancer Research made joint recommendations for broadening clinical trial criteria, to make results more representative.^6^ The LUNGevity Working Group has applied these recommendations to lung cancer.^7^ Prior cancers, meaning the exclusion of patients from a clinical trial protocol because a patient has had prior diagnosis of cancer unrelated to cancer for which a clinical trial is being considered, has been cited in both these initiatives as a common exclusion criterion of the need of change.

It is estimated that up to 14% of patients have prior history of cancer,^10^ which may be due to a common carcinogen exposure (for example cigarette smoking), genetic factors, or risks of second malignancies from prior anti‐cancer therapy. It is estimated that up to 18% of patients with non‐small cell lung cancer (NSCLC) are excluded from participating in clinical trials due to prior cancer.^11^ A potential reason for the exclusion of patients with prior cancer is the concern that prior cancer may affect prognosis. However, this was not the finding of two studies evaluating the survival of patients with incurable NSCLC^12^ and early‐stage NSCLC^13^ and prior malignancy using the SEER database.

There are, however, additional concerns regarding the inclusion of patients with prior cancer; If cancer recurs or progresses and needs treatment, the patient would need to be removed from the trial protocol, concern that prior cancer could complicate radiologic assessments or laboratory assessments if active or recurs, and concern that prior anticancer therapies could reduce tolerance of the investigational treatment under evaluation. While there have been a number of studies to address the survival of patients with NSCLC and a history of prior cancer, there have not been studies to look more comprehensively at these other factors.

This study consists of two complementary objectives. First, we sought to evaluate the frequency that systemic therapy clinical trials of incurable or metastatic NSCLC exclude patients with prior cancer, and secondly how frequently prior cancer would have impacted the validity of a clinical trial by evaluating a real‐world cohort of patients with NSCLC treated with full‐dose standard of care systemic therapy as a surrogate for a clinical trial population.

## 2 METHODS

This retrospective study was conducted at the Princess Margaret Cancer Center (PMCC), following Research Ethics Board approval. In this study, prior cancer was defined as a non‐lung cancer malignancy that was diagnosed prior to, or concurrent with (i.e., during the initial staging investigations), the diagnosis of incurable NSCLC. For the purposes of both Objective 1 and 2, incurable NSCLC was defined as stage IV, stage III not amenable to curative intent therapy, or recurrent disease after prior diagnosis of stage I‐III.

### 2.1 Objective 1

The first objective was to evaluate the proportion of interventional clinical trials of incurable NSCLC that still exclude patients with prior cancer, and to assess the difference or “stringency” of the exclusion of prior cancer across these trials. To evaluate the number of NSCLC clinical trials that exclude patients with prior history of cancer, the eligibility criteria of clinical trials registered on clinical trials.gov were reviewed. All trials of first‐line systemic therapy for the treatment of incurable NSCLC that were registered on clinicaltrials.gov between January 1st, 2014 and July 1st, 2017 were included. Data on the exclusion of patients with prior cancer, defined as any prior malignancy that was not an NSCLC, in addition to the date of trial registration and sponsor were extracted. Only trials that commented on the inclusion or exclusion of patients with prior cancer were included in the final analysis. For analysis purposes, the different prior cancer eligibility criteria extracted from clinicaltrials.gov, were classified into one of the three criteria by two reviewers MH and PB, as either pragmatic, stringent or moderate as follows:

*Pragmatic*: the protocol allows prior cancers with negligible risk of metastasis or death, or inactive cancers or any cancer not requiring treatment in the last 2 years.

*Stringent*: protocol excludes any prior cancer within the last 5 years, but can allow in situ malignancies for example in situ cervical cancer or DCIS.

*Moderate*: defined as not falling into the above categories.

Discrepancies in rating were resolved by consensus between MH and PB.

### 2.2 Objective 2

The second objective was to evaluate the potential impact of prior cancers on the validity of clinical trials by evaluating real‐world patient data from our institution. Specifically, we aimed to evaluate whether prior cancers could create challenges for investigators of NSCLC trials by interfering in the patient's ability to stay on trial or by affecting the measurement of commonly selected scientific objectives (such as response, progression or survival). In order to evaluate this, we created a list of surrogate events that, if they were to develop during a clinical trial, would result in a patient's exclusion from the trial, or would have interfered with the interpretation of a trial endpoint, (see definitions a‐f below). For example, evidence of active prior cancer on imaging would make the determination of the response rate of NSCLC to a trial intervention difficult. Similarly, evidence of recurrence of prior cancer could negatively impact survival irrespective of the NSCLC diagnosis. These events were defined a priori by PB and MH in discussion with experienced NSCLC trial principal investigators at our institution. A cohort of patients with incurable NSCLC, who were treated with full‐dose standard of care, first‐line systemic therapy at our Institution, were then evaluated for the development of events a‐f.

- Patient required concurrent (defined as during or after the diagnosis of incurable NSCLC) treatment of prior cancer with any systemic therapy, radiation, or surgery
- Evidence of active prior cancer on imaging, physical examination, or in the case of hematologic cancers on laboratory testing. If prior cancer had not been treated curatively, it was classified as active even if there were no radiologic, physical or laboratory findings.
- Increasing tumor marker of prior cancer at or after incurable NSCLC diagnosis
- Biopsy proven recurrence of prior cancer after the diagnosis of incurable NSCLC
- Death due to prior cancer
- Clinical uncertainty defined as the treating clinician documenting the possibility of the concurrent presence of prior cancer in the clinical chart

The frequency with which these events occurred was evaluated at baseline and at any time after the initiation of systemic therapy in a cohort of patients diagnosed with incurable NSCLC, and treated with systemic therapy between January 1^st^, 2010 and January 1^st^, 2016 at PMCC. All patients had a history of prior cancer, defined for the purposes of this study as invasive cancer, excluding non‐melanoma skin cancer, prior NSCLC, or carcinomas in‐situ. Eligible patients must have had biopsy‐confirmed cancer prior to a diagnosis of incurable NSCLC. Potentially eligible cases were identified from the PMCC Registry. The clinical chart was then reviewed and data extracted on patient demographics, treatments received, and if any of the events of interest occurred. In an attempt to include “fit” patients, only patients who received first‐line full dose, standard of care or clinical trial systemic therapy for NSCLC were included. For patients with multiple prior cancers, the most recent cancer was used to determine the timeframe between prior cancer and NSCLC diagnosis. Analysis of events a‐f defined above began on the date of the first assessment with a medical oncologist for metastatic or incurable NSCLC. Patients with an event present at the first assessment were analyzed separately from those who did not have an event at baseline.

Statistical considerations.

Kaplan–Meier survival curves were created for the overall survival (OS) of the study population. The exact method was used to calculate a 95% confidence interval on the proportion of patients who developed any of the events of interest.

## 3 RESULTS

Between January 1st, 2014 and July 1st, 2017 there were 359 trials of systemic therapy for incurable NSCLC registered on clinicaltrials.gov, of which 237 (66%) listed prior cancer in the eligibility criteria (Table 1). To ensure our events of interest were applicable to the primary endpoints of systemic therapy clinical trials, we determined that the proportion of trials that included OS or a radiologic‐based endpoint (e.g., progression‐free survival or response rate based on tumor measurement from CT scans) was 88%. In 12% of trials, the endpoint was one of a number of other endpoints including quality of life, pharmacokinetics, dose‐limiting toxicity, skeletal‐related events or biomarker‐based endpoints. However, similarly, a recurrence of prior cancer would be expected to interfere with the measurement of these endpoints. Of the trials that listed prior cancer in the eligibility criteria, exclusion criteria were similarly distributed between the pragmatic, moderate, and stringent categories (30%, 31%, and 39%, respectively). One hundred and twenty‐four (52%) of criteria did not specify a required timeframe from the diagnosis of prior cancer to the NSCLC diagnosis in which patients would be eligible for enrollment. For trials that did specify a minimum cancer‐free interval for prior cancers, five years was most common at 83 (35%). A minority of trials (24%) allowed leeway on the part of the investigator to determine which patients with prior cancers could be enrolled, and 15% of protocols excluded only those patients concomitantly on another anti‐cancer therapy. Numerically, there was no association between the year the trial was registered on clinicaltrials.gov and the stringency of prior cancer exclusion criteria; however, there was a trend toward industry‐sponsored trials being more restrictive of patients with prior cancers (Pragmatic exclusion criteria in 21% vs. 37%). Due to the fact that 33% of trials did not list prior cancer in the eligibility criteria this association did not undergo statistical testing.

**TABLE 1:** Clinical trial characteristics on clinicaltrials.gov

Variable		Result N (%)	*p*‐value for strength of exclusion criteria
Total number of interventional trials in incurable NSCLC on clinicaltrials.gov that were reviewed		359	
Total number of trials with prior cancer listed in the eligibility criteria		237	
Strength of exclusion criteria^a^	Pragmatic	70 (30)	
	Moderate	74 (31)	
	Stringent	93 (39)	
Year of registration^a^	2014	52 (22)	*p *= 0.86
	2015	76 (32)	
	2016	71 (30)	
	2017	38 (16)	
Industry sponsored^a^		111 (47)	*p *= 0.02^b^
Stated timeframe of prior cancer exclusion			
0.5 years		2 (1)	
1 year		5 (2)	
2 years		23 (10)	
5 years		83 (35)	
Not specified		124 (52)	
Number allowing investigator assessment		56 (24)	
Number restricting only if concomitant anti‐cancer therapy needed now or likely in future		35 (15)	

^a^ From the cohort of trials with prior malignancy listed in eligibility criteria (n=237)

^b^ Industry‐sponsored studies were less likely to have pragmatic exclusion criteria

**FIGURE 1:**
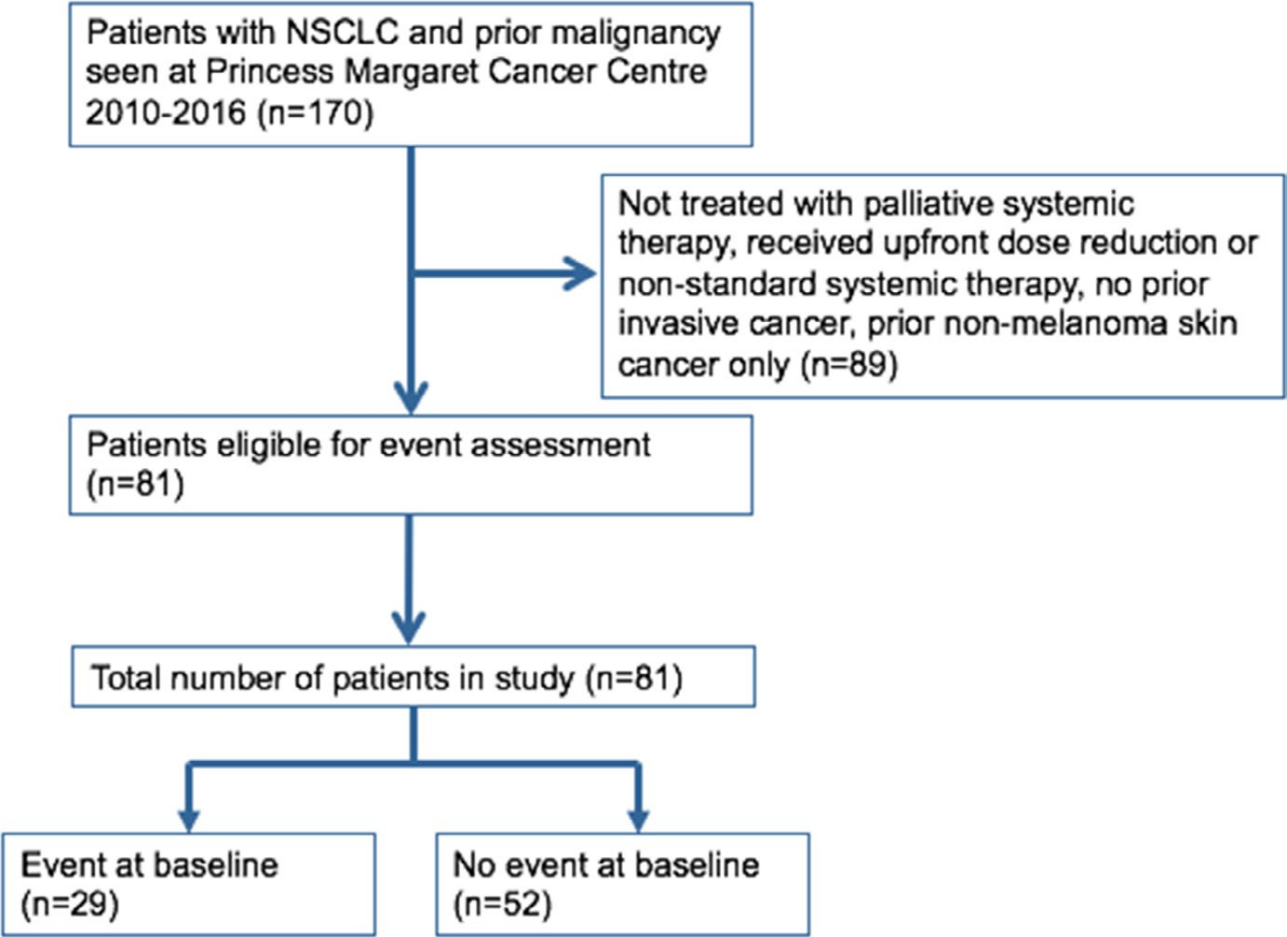
CONSORT diagram

**FIGURE 2:**
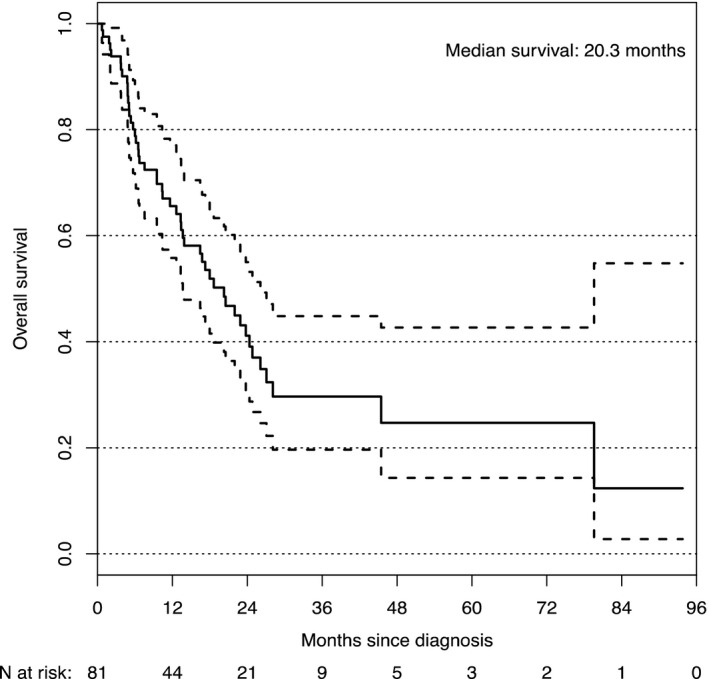
Kaplan–Meier plot of the overall survival of Stage IV NSCLC patients eligible for event assessment in PMCC cohort

Between January 1st, 2010 and January 1st, 2016 there were 852 patients with incurable NSCLC commenced on first‐line systemic therapy at our institution, of which 170 (20%) had a history of prior cancer excluding non‐melanoma skin cancer, prior NSCLC, or carcinomas in‐situ. Eighty‐one patients with prior invasive cancer received full dose, standard of care, or clinical trial, first‐line systemic therapy for incurable NSCLC (Figure 1 and Table 2). Of this group, the median age was 67 years, 44% were male and 66% were current or past smokers. The most common histology was adenocarcinoma and activating *EGFR* mutations or *ALK* translocations were seen in 31% and 4%, respectively. The median OS of all patients in the study was 20 months (Figure 2). The majority of patients (83%) had no prior treatment of NSCLC before starting systemic therapy. The most common types of prior cancer were breast and prostate cancer, seen in 20% and 19%, respectively, but there was a broad representation of primary sites (Table 2). Of all prior cancers, 80% had been treated curatively with a median time between prior cancer and the NSCLC diagnosis of 82 months. At any point during their treatment course, 26% participated in systemic therapy clinical trials.

**TABLE 2:** Clinical characteristics of NSCLC patients with prior cancers

Variable	Description	N (%) unless specified
Overall		N = 81
Age	Median years (range)	67 (41–94)
Sex	Male	36 (44)
Smoking History	Current smoker	12 (15)
	Ex‐smoker	41 (51)
	Never‐smoker	28 (34)
Pack years	Median (range)	20 (0–100)
Histology	Adenocarcinoma	64 (79)
	Squamous cell	13 (16)
	Large cell	1 (1)
	Other	3 (4)
*EGFR* mutation	Mutation	25 (31)
	Wildtype	26 (32)
	unknown	30 (37)
*ALK* translocation	Translocation	3 (4)
	Wildtype	37 (46)
	unknown	41 (51)
PDL−1 staining	<1%	1 (1)
	1–49%	2 (3)
	≥50%	1 (1)
	unknown	77 (95)
Prior NSCLC Treatment	Chemotherapy + Radiation	3 (4)
	Surgery	2 (3)
	Surgery + Chemotherapy	4 (5)
	Tri‐modality	5 (6)
	No prior treatment	67 (83)
Clinical trial participation	(any line)	21 (26)
Number of prior cancers	1	67 (83)
	2	13 (16)
	≥3	1 (1)
Prior cancers	Breast	16 (20)
	Prostate	15 (19)
	Colon	6 (7)
	Melanoma	2 (3)
	Gynecological	7 (9)
	Head and neck	6 (7)
	Bladder	3 (4)
	Hematological	7 (9)
	Non‐colon GI	3 (4)
	Renal	1 (1)
	Thyroid	10 (12)
	Other	5 (6)
Received curative‐intent treatment for prior cancer		65 (80)
Time between prior cancer and NSCLC diagnosis^a^	Median (range) months	82 (0.3–546)

^a^ For patients with multiple prior cancers most recent used.

**FIGURE 3:**
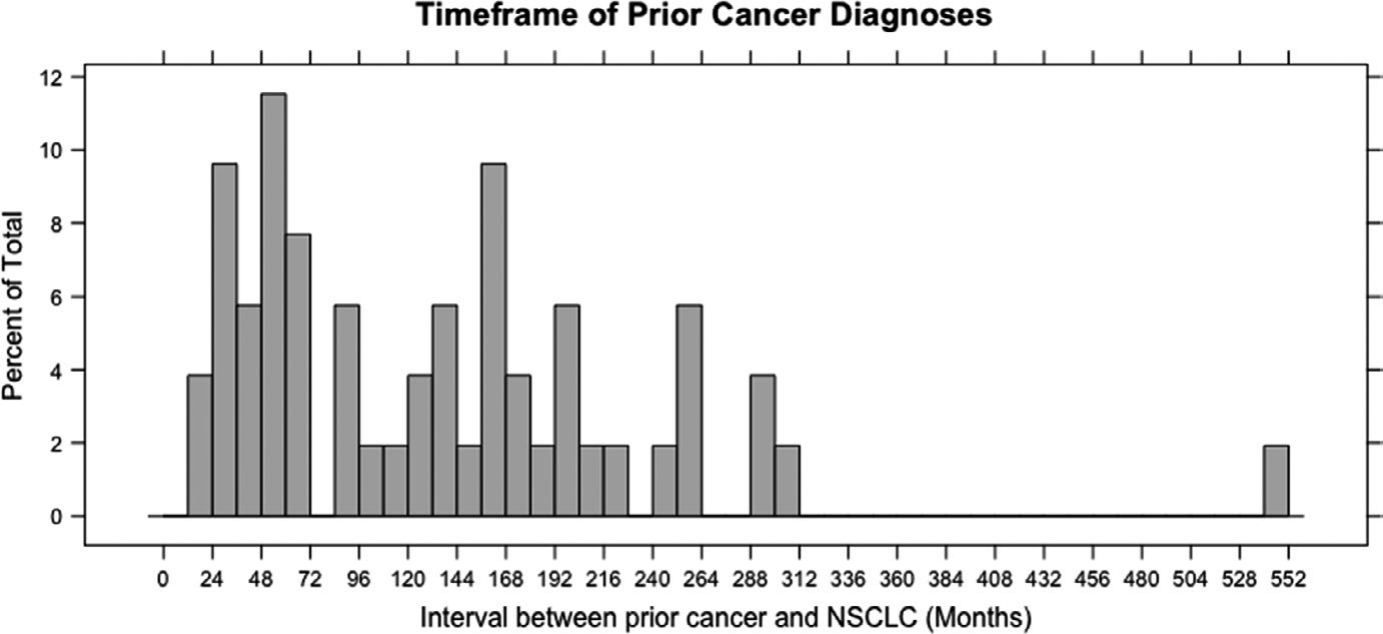
Timeframe of Prior Cancer Diagnoses for Patients without Baseline Event reported as a Percentage of all prior cancers

The analysis of patients who developed an event (a–f), was divided into those patients who had an event already at the baseline assessment with a medical oncologist for incurable NSCLC, versus those free of an event at baseline assessment (Table 3). There were 29 patients with any event at the baseline visit. The most common events present at the baseline assessment included evidence of active prior cancer, clinician uncertainty as to the presence of prior cancer, or receiving anti‐cancer therapy for prior cancer (18, 14, and 9 patients, respectively). In the subgroup of 52 patients with no event at baseline, none developed an event during a median follow‐up of 45 months; no patients had evidence of a recurrence of prior cancer during the treatment and follow‐up for the NSCLC, or died from prior cancer (Table 3). In this group, 31% had prior cancer within the past 5 years (Figure 3). However, two patients in this subgroup developed a new primary cancer after baseline, in one case a stage 3 colon cancer and in another case, imaging suggested a pancreatic neuroendocrine tumor, unrelated to their prior malignancy. The outcomes of four patients with non‐curatively treated prior cancers, but no other baseline events were also evaluated; all four patients received full‐dose first‐line chemotherapy for the incurable NSCLC, none died from or required treatment for the other malignancy, and there was no evidence of the other malignancy on scans that impacted the interpretation of response to the NSCLC systemic therapy.

**TABLE 3:** Frequency (and percentage) of events of interest

Events of interest	N (%) All patients	N Excluding patients with any event at baseline assessment^a^
Total number of patients with one or more events of interest at or after baseline	29 (36)	0^b^
Number requiring anti‐cancer therapy for prior cancer (radiation, surgery, systemic therapy)	13 (16)	0
At baseline	9	
After baseline	4	
Evidence of active prior cancer on imaging, physical examination, or laboratory testing	19 (23)	0
At baseline	18	
After baseline	1	
Rising tumor markers of prior cancer	3 (4)	0
At baseline	0	
After baseline	3	
Biopsy revealing a recurrence of prior cancer	4 (5)	0
At baseline	0	
After baseline	4	
Death due to another cancer	2 (2)	0
At baseline	0	
After baseline	2	
Physician uncertainty defined as the possibility of concurrent prior cancer and NSCLC on imaging	16 (20)	0
At baseline	14	
After baseline	2	

Note

Allowing patients with indolent cancers added four additional patients, none of which developed an event.

^a^ Total number of patients in this subgroup = 52.

^b^ Two patients developed new primaries during follow‐up.

## 4 DISCUSSION

NSCLC incidence increases with older age and a history of smoking.^14^ These risk factors are shared by many other cancers, and consequently, patients with NSCLC have a high rate of prior cancers.^15^ This can reduce enrollment on clinical trials, which traditionally exclude patients with a history of prior cancer. Trial sponsors may exclude patients with prior cancer because of potential prognostic implications, or concern that it may impact safety or one of the clinical trial endpoints. Despite the frequency that patients with prior cancer are excluded from clinical trials, there are little data to support the exclusion or to inform on the optimal eligibility criteria for clinical trial protocols. A number of studies have evaluated the survival of patients with lung cancer and a history of prior malignancy,^12^, ^13^ but we sought to examine additional factors that could impact the ability to deliver the investigational treatment, and common clinical trial endpoints, from a cohort of patients with incurable NSCLC treated with full‐dose first‐line systemic therapy, as a surrogate clinical trial population.

In our study, prior cancer proved to be common exclusion criteria in incurable NSCLC clinical trials of first‐line systemic therapy. Furthermore, there was a wide range of exclusions related to prior cancers, although a blanket exclusion of 5 years from prior cancer was common. It is acknowledged that in a third of trials, the exclusion criteria did not refer to prior cancer in the criteria listed in the clinicaltrials.gov listings. This could be because prior cancer was not an exclusion or that the full inclusion and exclusion criteria were not posted. However, even if it was the former, a majority of trials still exclude patients with prior cancers.

Laccetti et al. analyzed Eastern Oncology Cooperative Group (ECOG) trial protocols, and found 80% of trials contained prior cancer exclusion, with 43% restricting patients with prior cancer in the last 5 years.^11^ They found that prior cancer was excluded more often and more broadly in older clinical trials and phase 2 and 3 studies compared to phase 1, and in studies with survival as the primary endpoint.^11^ In the cohort of trials that included details of prior cancer eligibility on clinicaltrials.gov, we found industry‐sponsored trials were less likely to have pragmatic criteria relating to prior cancers, and although there was no difference in stringency by year of trial registration, we only analyzed trials starting in 2014.

In our study, we found that 20% of patients with NSCLC had prior cancer, similar to the 18.7% reported in a previous SEER analysis.^15^ Patients in our cohort had a median OS of 20 months, which compares favorably to historical data of patients with incurable NSCLC.^16^ In a study by Laccetti et al, using the SEER database, patients with advanced NSCLC and prior cancer had an improved all‐cause and lung cancer‐specific survival compared with NSCLC patients but without prior cancer.^12^ In a study of patients with early‐stage lung cancer and prior cancer, similar results were reported with no reduction in survival for patients with prior cancer.^13^ One possible explanation is that this group of patients would have frequent imaging and follow‐up for their prior cancers, which may lead to the earlier discovery of NSCLC when it is at a lower disease burden and more amenable to treatment. Similar findings have been reported in other malignancies. In a study in gastrointestinal cancer^17^ there was an improved colorectal cancer‐specific survival in patients with stage IV disease and prior non‐hematological malignancy and no significant change in OS compared with patients with colorectal cancer but no prior cancer; and prior cancer did not lead to an inferior survival in patients with nasopharyngeal carcinoma.^18^

We aimed to look at factors other than survival that may impact trial conduct or impact. We defined six criteria of interest and in a cohort of patients treated with full‐dose first‐line systemic therapy, we found that no patients developed one of these pre‐specified events during follow‐up, provided they did not already have an event at their baseline assessment visit.

with a medical oncologist. Of the patients in this group, 31% had prior cancer in the previous five years, indicating that a significant number would have been excluded using the common 5‐year exclusion.

ASCO and Friends of Cancer Research recently released guidelines on modernizing clinical trial eligibility criteria.^19^ The guidelines recommended including patients with prior malignancies in clinical trials, especially if they fulfill the following criteria: the risk of the prior malignancy interfering with either safety or efficacy endpoints is very low; all treatments of the prior malignancy were completed at least two years before registration and the patient has had no further evidence of disease. The LUNGevity Working Group applied these recommendations to advanced lung cancer trials^7^ and recommended for lung cancer‐specific metastatic or recurrent disease trials that there be no restriction for prior malignancy, except for invasive active malignancy requiring ongoing therapy.

Our findings support these recommendations by providing evidence of clinical factors that are associated with a low likelihood of prior cancers interfering with either trial safety or efficacy endpoints in NSCLC patients. Although we did not specify a strict time cut‐off for the treatment of prior cancer, only 4% of the final cohort had treatment of their prior cancer within the last two years. The ASCO and Friends of Cancer Research guidelines also call for extending eligibility criteria to all patients with concurrent malignancy in clinical trials, providing that it is clinically stable and does not require tumor‐directed treatment. Due to the small numbers of patients, we are unable to conclude patients with concurrent cancers that are clinically stable; however, this is a patient subset that deserves further study.

There are several limitations to our study. First, this is a retrospective observational study that carries the inherent limitations of such an approach. The size of the final cohort of patients was small; however, calculated 95% confidence intervals were narrow. Further, patients may have been ineligible for clinical trial participation for reasons unrelated to prior cancer, which were not captured in this study. However, the selection of patients who received full dose, the standard of care systemic therapy helped mitigate this concern. In fact, 26% of patients in this cohort did go on a clinical trial at some point during follow‐up. Patients with prior cancer were located using our institutional cancer registry, which does not track all prior cancers, such as past non‐melanoma skin cancer or in‐situ cancers. Finally, the eligibility criteria reported on clinicaltrials.gov may not reflect the eligibility criteria in the final protocol of some clinical trials.

If validated in larger data sets, the following criteria could be considered to select patients with prior cancer for the clinical trial: no evidence of prior cancer on imaging, physical exam or laboratory studies, normal tumor marker levels for prior cancer, not on anti‐cancer therapy for prior cancer, and no clinician uncertainty as to the presence of concomitant prior cancer and NSCLC. Our results would suggest patients meeting these criteria have a low likelihood of having prior cancer affect clinical trial results.

## CONFLICT OF INTEREST

There are no relevant conflict of interests identified by the authors.

## PRIOR PRESENTATION

Herman, M., Liu, Z., Liu, G., Leighl, N., Shepherd, F., Bradbury, P. Investigating the effect of prior malignancy on non‐small cell lung cancer trial eligibility [abstract]. In: 19^th^ World Conference on Lung Cancer; 2018 Sept 23–26; Toronto, ON. P2.01.

## Data Availability

The data that support the findings of this study are available on request from the corresponding author. The data are not publicly available due to privacy or ethical restrictions.

## References

[1] Institute of Medicine (US) . Forum on Drug Discovery, Development, and Translation. Transforming Clinical Research in the United States: Challenges and Opportunities: Workshop Summary. Washington (DC): National Academies Press (US); 2010; 6, Clinical Trials in Cancer. Available from: https://www.ncbi.nlm.nih.gov/books/NBK50895/ 21210556

[2] Unger JM , Cook E , Tai E , Bleyer A . The role of clinical trial participation in cancer research: barriers, evidence, and strategies. Am Soc Clin Oncol Educational Book. 2016;35:185‐198. 10.1200/EDBK_156686. 27249699 PMC5495113

[3] Fuks A , Weijer C , Freedman B , Shapiro S , Skrutkowska M , Riaz A . A study in contrasts: eligibility criteria in a twenty‐year sample of NSABP and POG clinical trials. National Surgical Adjuvant Breast and Bowel Program. Pediatric Oncology Group. J Clin Epidemiol. 1998;51(2):69‐79. 10.1016/s0895-4356(97)00240-0. 9474067

[4] Van Spall HGC , Toren A , Kiss A , Fowler RA . Eligibility criteria of randomized controlled trials published in high‐impact general medical journals: a systematic sampling review. JAMA. 2007;297(11):1233‐1240. 10.1001/jama.297.11.1233. 17374817

[5] Duma N , Kothadia SM , Azam TU , et al. Characterization of comorbidities limiting the recruitment of patients in early phase clinical trials. Oncologist. 2019;24(1):96‐102. 10.1634/theoncologist.2017-0687. 30413668 PMC6324635

[6] Kim ES , Bruinooge SS , Roberts S , et al. Broadening eligibility criteria to make clinical trials more representative: american society of clinical oncology and friends of cancer research joint research statement. J Clin Oncol. 2017;35(33):3737‐3744. 10.1200/JCO.2017.73.7916. 28968170 PMC5692724

[7] Bonomi P , Blumenthal G , Ferris AS , et al. Making lung cancer clinical trials more inclusive: recommendations for expanding eligibility criteria. J Thorac Oncol. 2018;13(6):748‐751. 10.1016/j.jtho.2018.02.013. 29793646

[8] Torre LA , Siegel RL , Jemal A . Lung cancer statistics. Adv Exp Med Biol. 2016;893:1‐19. 10.1007/978-3-319-24223-1_1. 26667336

[9] Arbour KC , Riely GJ . Systemic therapy for locally advanced and metastatic non‐small cell lung cancer: a review. JAMA. 2019;322(8):765‐771. 10.1001/jama.2019.11058. 31454018

[10] VanderWalde AM , Hurria A . Second malignancies among elderly survivors of cancer. Oncologist. 2011;16(11):1572‐1581. 10.1634/theoncologist.2011-0214. 22042787 PMC3233292

[11] Gerber DE , Laccetti AL , Xuan L , Halm EA , Pruitt SL . Impact of prior cancer on eligibility for lung cancer clinical trials. J Natl Cancer Inst. 2014;106(11): 10.1093/jnci/dju302. PMC4271029 25253615

[12] Laccetti AL , Pruitt SL , Xuan L , Halm EA , Gerber DE . Effect of prior cancer on outcomes in advanced lung cancer: implications for clinical trial eligibility and accrual. J Natl Cancer Inst. 2015;107(4): 10.1093/jnci/djv002. PMC4402360 25667420

[13] Pruitt SL , Laccetti AL , Xuan L , Halm EA , Gerber DE . Revisiting a longstanding clinical trial exclusion criterion: impact of prior cancer in early‐stage lung cancer. Br J Cancer. 2017;116(6):717‐725. 10.1038/bjc.2017.27. 28196065 PMC5355931

[14] Molina JR , Yang P , Cassivi SD , Schild SE , Adjei AA . Non‐small cell lung cancer: epidemiology, risk factors, treatment, and survivorship. Mayo Clin Proc. 2008;83(5):584‐594. 10.4065/83.5.584. 18452692 PMC2718421

[15] Murphy CC , Gerber DE , Pruitt SL . Prevalence of prior cancer among persons newly diagnosed with cancer: an initial report from the surveillance, epidemiology, and end results program. JAMA Oncol. 2018;4(6):832‐836. 10.1001/jamaoncol.2017.3605. 29167866 PMC6370034

[16] Bradley CJ , Yabroff KR , Mariotto AB , Zeruto C , Tran Q , Warren JL . Antineoplastic treatment of advanced‐stage non‐small‐cell lung cancer: treatment, survival, and spending (2000 to 2011). J of Clin Oncol. 2017;35(5):529‐535. 10.1200/JCO.2016.69.4166. 28045621 PMC5455316

[17] Smyth EC , Tarazona N , Peckitt C , et al. Exclusion of gastrointestinal cancer patients with prior cancer from clinical trials: is this justified? Clin Colorectal Cancer. 2016;15(2):e53‐e59. 10.1016/j.clcc.2015.11.003. 26747392

[18] Wang Y‐Q , Lv J‐W , Tang L‐L , et al. Effect of prior cancer on trial eligibility and treatment outcomes in nasopharyngeal carcinoma: implications for clinical trial accrual. Oral Oncol. 2019;90:23‐29. 10.1016/j.oraloncology.2019.01.023. 30846172

[19] Lichtman SM , Harvey RD , Damiette Smit M‐A , et al. Modernizing clinical trial eligibility criteria: recommendations of the american society of clinical oncology‐friends of cancer research organ dysfunction, prior or concurrent malignancy, and Comorbidities Working Group. J Clin Oncol. 2017;35(33):3753‐3759. 10.1200/JCO.2017.74.4102. 28968172

